# Ion temperature anisotropy across a magnetotail reconnection jet

**DOI:** 10.1002/2015GL065168

**Published:** 2015-09-18

**Authors:** H. Hietala, J. F. Drake, T. D. Phan, J. P. Eastwood, J. P. McFadden

**Affiliations:** ^1^ The Blackett LaboratoryImperial CollegeLondonUK; ^2^ Department of Physics, the Institute for Physical Science and Technology and the Joint Space InstituteUniversity of MarylandCollege ParkMarylandUSA; ^3^ Space Science LaboratoryUniversity of CaliforniaBerkeleyCaliforniaUSA

**Keywords:** magnetic reconnection, magnetotail, temperature anisotropy, ion heating

## Abstract

A significant fraction of the energy released by magnetotail reconnection appears to go into ion heating, but this heating is generally anisotropic. We examine ARTEMIS dual‐spacecraft observations of a long‐duration magnetotail exhaust generated by antiparallel reconnection in conjunction with particle‐in‐cell simulations, showing spatial variations in the anisotropy across the outflow far (>100*d*_*i*_) downstream of the X line. A consistent pattern is found in both the spacecraft data and the simulations: While the total temperature across the exhaust is rather constant, near the boundaries *T*_*i*,||_ dominates. The plasma is well above the firehose threshold within patchy spatial regions at |*B*_*X*_|∈[0.1,0.5]*B*_0_, suggesting that the drive for the instability is strong and the instability is too weak to relax the anisotropy. At the midplane ( $|B_{X}|≲0.1B_{0}$), *T*_*i*,⊥_>*T*_*i*,||_ and ions undergo Speiser‐like motion despite the large distance from the X line.

## Key Points

- Parallel temperature peaks at the edges, perpendicular at the neutral plane
- Firehose instability threshold is greatly exceeded, indicating strong driving
- Speiser‐like motion persists far from X line despite large fluctuations

## 1 Introduction

Magnetic reconnection redistributes energy by releasing magnetic energy into particle energies—high speed bulk flows, heating, and particle acceleration. With near‐Earth in situ observations, we have access to three main parameter regimes: the solar wind, magnetopause, and magnetotail. Ion heating, in terms of an increase in temperature (obtained from the second velocity moments of the distribution function), has been systematically studied in reconnection exhausts in the solar wind [*Drake et al.*, 2009; *Enžl et al.*, 2014] and at the magnetopause [*Phan et al.*, 2014], where the available magnetic energies per particle $B_{\text{in}}^{2}/\mu_{0}n_{\text{in}}=m_{i}V_{\text{A,in}}^{2}$ are around 10^1^–10^2^ eV and 10^2^–10^4^ eV, respectively. Reconnection jets in the magnetotail, where $m_{i}V_{\text{A,in}}^{2}$ is higher (∼10^4^−10^5^ eV), the inflow plasma beta *β*_in_ is very low, and the boundary conditions are typically antiparallel and symmetric, offer another regime for investigation. Many of the earlier observations have been recently summarized by, e.g., *Paschmann et al.*[2013] and *Fuselier and Lewis* [2011].

Ion heating could arise from the interpenetration of the two particle populations entering the exhaust from either side of the current sheet [*Cowley*, 1982]. These counterstreaming beams have been observed at the magnetopause [*Gosling et al.*, 1990], in the magnetotail [*Hoshino et al.*, 1998], solar wind [*Gosling et al.*, 2005], and magnetosheath [*Phan et al.*, 2007]. The resulting temperature anisotropy varies across the exhaust, as shown by the simulations of, e.g., *Liu et al.* [2012]. Previous observations [e.g., *Hoshino et al.*, 1997; *Gosling et al.*, 2005; *Phan et al.*, 2007; *Wu et al.*, 2013; *Phan et al.*, 2014] show that the plasma temperature parallel to the magnetic field is generally larger than the perpendicular temperature. However, quantifying the spatial variations in the turbulent exhaust using short duration single spacecraft observations is difficult.

A temperature anisotropy where *T*_||_>*T*_⊥_ is important because it counteracts the magnetic tension force that accelerates the jet, and it supports a long current sheet [e.g., *Rich et al.*, 1972; *Cowley*, 1978; *Le et al.*, 2014]. If the temperature anisotropy is large enough, namely, *α* = (*β*_||_−*β*_⊥_)/2 > 1, the plasma will become firehose unstable [e.g., *Liu et al.*, 2012]. This leads us to ask the question whether the instability limits the anisotropy. Temperature anisotropy also governs the structure of the exhaust boundary: Parametric studies and simulations [e.g., *Lyu and Kan*, 1986; *Liu et al.*, 2012] indicate that large *T*_||_/*T*_⊥_ tends to suppress the formation of slow shocks. Interestingly, simulations [*Arzner and Scholer*, 2001; *Higashimori and Hoshino*, 2012; *Liu et al.*, 2012] do not agree on whether or not the anisotropy decreases at large distances ( $≳\;100\;d_{i}$) from the X line.

The dynamics of interpenetrating ions vary depending on the exhaust geometry—opening angle, distance to the X line, and to the reconnection front—as the curvature of the field line changes [e.g., *Nakamura et al.*, 1998]. The particle motion is controlled by parameter $\kappa \;=\;\sqrt{R_{\min}/r_{\text{L,max}}}\;=\;\Omega_{\text{ci,N}}/\omega_{\text{bi}}$, where *R*_min_ is the minimum radius of field line curvature, *r*_L,max_ the maximum Larmor radius of the particles, *Ω*_ci,N_ the gyrofrequency in the field component normal to the current sheet, and *ω*_bi_ the ion bounce frequency across the current sheet [e.g., *Buechner and Zelenyi*, 1989]. The motion is characterized as Speiser for small *κ*, chaotic for *κ* ∼ 1, and magnetized for *κ* > 1. In the Speiser regime [*Speiser*, 1965] the motion is a combination of rapid bouncing across the field reversal region and slow rotation around *B*_*N*_. Thus, if the field reversal region within the (antiparallel) exhaust is thin, ions meander near it with a range of *v*_⊥_ values [*Drake et al.*, 2009] and previous simulations show *T*_⊥_>*T*_||_ at the midplane [*Nakamura et al.*, 1998; *Lottermoser et al.*, 1998]. However, this motion is reversible so that the ions revert back to a beam upon exiting this region [*Drake et al.*, 2009]. Consequently, Speiser orbits preserve (some of) the temperature information of the inflowing populations, and simulations by *Higashimori and Hoshino* [2015] suggest that this property leads to a clear *β*_in_ dependence in the excitation and damping of the exhaust fluctuations. *κ* increases with increasing distance to the X line because Rmin increases as the exhaust widens. Meandering Speiser‐like ion motion has recently been observed within the ion diffusion region [*Nagai et al.*, 2015], but how far downstream does this regime extend?

Here we present a detailed study of a long‐duration, antiparallel, symmetric magnetotail reconnection exhaust using the two ARTEMIS spacecraft [*Angelopoulos*, 2011] at 52 and 59 *R*_*E*_ downtail (*R*_*E*_=6371 km, Earth's radius). We compare the observations to the large scale particle‐in‐cell (PIC) simulation previously analyzed by *Liu et al.* [2012]. We address (i) the ion temperature increase for large *V*_A,in_ and low *β*_in_ conditions, (ii) ion temperature anisotropy—its spatial variations and firehose instability, and (iii) the underlying ion dynamics and the extent of Speiser regime.

## 2 Data, Methods, and Overview

**Figure 1:**
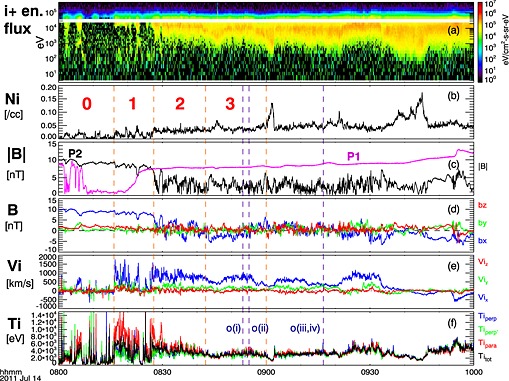
Overview of observations from P2 unless noted. (a) Ion energy spectrogram (electrostatic analyzer and solid state telescope), (b) ion density, (c) magnetic field magnitude (P1 in magenta) and (d) its GSM components, (e) ion velocity components, and (f) ion temperature. The orange dashed lines mark the different analysis intervals (0–3) used for Figure 3. The dark blue dashed lines o(i–iv) mark the times when the distributions shown in Figure 4 were taken.

Figure 1 shows the overview of the spacecraft observations from 08:00 to 10:00 UT on 14 July 2011. Further details of the data and instruments are given in the supporting information. P2 was located near midnight at [−52.6,3.1,1.8] *R*_*E*_, with P1 duskwards and tailward from it at [−59.0,15.4,−3.1] *R*_*E*_. During the interval under consideration, P1 and P2 moved ∼1 *R*_*E*_ in the −*Y*_GSM_ direction while retaining their separation. At the beginning of the event, the plasma sheet moved northwards so that P1 moved from the plasma sheet into the southern lobe (*B*_*X*_<0) and P2 from the northern lobe (*B*_*X*_>0) into the plasma sheet at 08:27:30 UT (Figure 1c), where it observed an earthward reconnection jet with an average speed of ∼700 km/s and maximum speed exceeding 1500 km/s (Figure 1e).

We have divided the event into four analysis intervals: the first encounter with the boundary (interval 0, 08:00:00–08:16:00 UT); the exhaust boundary (interval , 08:16:00–08:27:30 UT); the first 15 min in the exhaust proper while the density remained stable (interval 2, 08:27:30–08:42:30 UT); and the following observations until the appearance of the large density peak and the increase of P1 magnetic field magnitude above 8 nT (interval 3, 08:42:30–09:00:00 UT).

The lobe magnetic field strength was 8–10 nT. *B*_*Z*_ in the exhaust (Figure 1d) was on average small but positive, as appropriate for an earthward reconnection jet. *B*_*Y*_ seen by P2 in the lobe was very small, typically around 0.5 nT or less (∼0.05*B*_0_), suggesting that the guide field was small and reconnection involves essentially antiparallel fields. The plasma densities (Figure 1b) varied slowly over time: in the exhaust (P2) the density was typically 0.04–0.05 cm^−3^, with some intervals where it increased to 0.07 and 0.14cm^−3^. In the lobe (P1 and P2) the density was mostly low, $≲0.03\;{\text{cm}}^{-3}$. In the main exhaust after 08:30 UT the ion temperature (Figure 1f) was ∼4 keV, but near the exhaust edge the parallel temperature was as high as ∼15 keV and the total ion temperature enhanced.

In the exhaust the ion inertial length *d*_*i*,exhaust_=*c*/*ω*_pi_ was 0.16–0.18 *R*_*E*_ (*n* = 0.04 − 0.05cm^−3^). For temperatures of 4 keV and magnetic field values of 8, 5, and 2 nT, the ion gyroperiod $f_{\text{ci}}^{-1}$ was 8.2, 13, and 33 s, and the Larmor radius *r*_*L*_ was 0.18, 0.29, and 0.72 *R*_*E*_. To estimate the upstream Alfvén speed, we use *B*_0_=8 nT and $n_{0}=0.02\;{\text{cm}}^{-3}$, which is also consistent with a compression ratio of two typical for antiparallel symmetric reconnection simulation [e.g., *Liu et al.*, 2012], giving *V*_A,in_∼1200 km/s. The upstream ion plasma beta was ≲0.05. Using two spacecraft timing and assuming planarity, no tilt, and purely northward motion we can estimate the plasma sheet thickness to be ∼2 *R*_*E*_∼10*d*_i,exhaust_.

**Figure 2:**
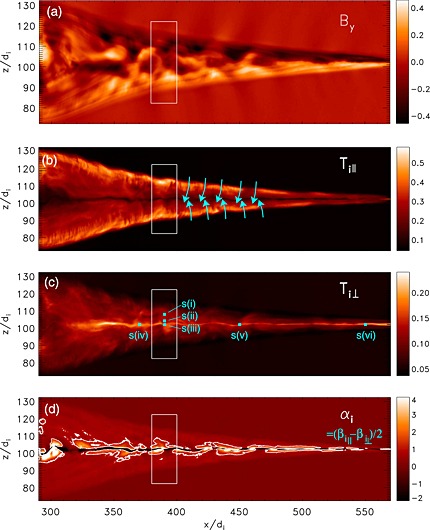
Temperature anisotropy in the 2.5D particle‐in‐cell simulation. (a) Out‐of‐the‐plane magnetic field, (b and c) ion parallel and perpendicular temperatures, and (d) the ion anisotropy *α*_*i*_=(*β*_*i*,||_−*β*_*i*,⊥_)/2. The firehose condition *α*_*i*_=1 is indicated with a white contour in Figure 2d. The cyan arrows in Figure 2b illustrate the two inflowing populations. The white rectangle shows the region where the vertical cuts shown in Figure 3 were made. The cyan squares in Figure 2c indicate where the distributions shown in Figure 4 were taken.

We compare these observations with 2.5D PIC simulation results shown in Figure 2, obtained using the P3D code [*Zeiler et al.*, 2002]. The simulation run [*Liu et al.*, 2012] had *m*_*i*_/*m*_*e*_=25, *c* = 15*V*_A_, *β*_in_=0.2, *T*_*i*_/*T*_e_=1, 100 particles per cell, box size 819.2*d*_*i*_×409.6 *d*_*i*_, and an initial double Harris sheet configuration. At 350 $\;\Omega_{\text{ci}}^{-1}$ into the run, we uniformly sample points along 100 vertical cuts within a 20 *d*_*i*_ wide strip across the exhaust at ∼175 *d*_*i*_ (∼105 *d*_*i*,exhaust_) away from the X line (white box in Figure 2). The exhaust width at this distance is ∼13 *d*_*i*,exhaust_, similar to the ARTEMIS event.

## 3 Results

**Figure 3:**
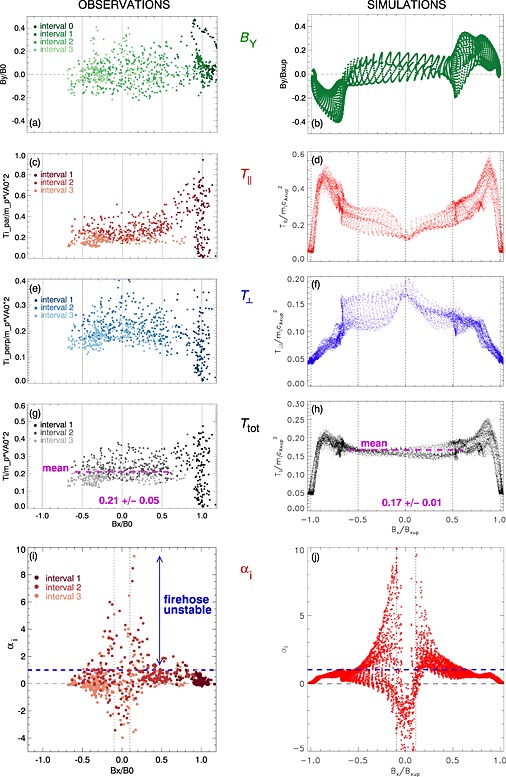
Profiles of observed and simulated quantities as functions of estimated distance to the neutral plane. (a and b) Out‐of‐the‐plane magnetic field, (c and d) ion parallel and (e and f) perpendicular temperatures, (g and h) the total ion temperature, and (i and j) ion temperature anisotropy *α*_*i*_=(*β*_*i*,||_−*β*_*i*,⊥_)/2. The blue dashed line in Figures 3i and 3j indicates the firehose instability threshold *α*_*i*_=1 (assuming cold/isotropic electrons). The magenta dashed lines in Figures 3g and 3h indicate the mean total ion temperature within |*B*_*X*_|<0.6*B*_0_.

Figure 3 displays the out‐of‐the‐plane magnetic field and ion temperature mapped against the reconnecting field (a proxy for the distance to neutral plane), with spacecraft measurements on the left and simulation cuts on the right. We have indicated the time evolution of the observations with different shading. The spacecraft did not traverse the whole plasma sheet, and thus, we have no observations on the left hand side near *B*_*X*_/*B*_0_∼−1. For the simulated profiles we use *B*_*x*,up_ taken just upstream of the exhaust, which was 0.85 of the original upstream *B*.

Figure 2a shows that the out‐of‐the‐plane magnetic field is well organized to positive and negative near the X line ( $≲\;80d_{i}$) but becomes turbulent farther downstream. Considering the observed *B*_*Y*_ profile (Figure 3a) and the simulated one (Figure 3b), we see that these characteristic positive‐negative Hall field signatures [e.g., *Mandt et al.*, 1994; *Eastwood et al.*, 2010] are present only at the very edges of the exhaust, (note *B*_*Y*_∼+4 nT ∼0.5*B*_0_ at 08:06 UT, Figure 1d), while the midexhaust had a rather uniform distribution of *B*_*Y*_ fluctuations across it. The negative *B*_*Y*_ at *B*_*x*_∼0.6*B*_*x*,up_ (Figure 3b) corresponds to a transient local structure in the simulation. Based on the absence of the characteristic Hall field within midexhaust and the similarity with the simulation results of Figure 3b and of *Higashimori and Hoshino* [2012], we conclude that the spacecraft was far downstream of the X line, probably >100*d*_*i*_.

The shape of the observed temperature profiles is in good agreement with the simulations (Figures 3c–3h). At the edge of the exhaust, *T*_||_ increases sharply. It then decreases, going down to about a third of its peak value at the neutral plane. *T*_⊥_ also increases at the edge of the exhaust, although this occurs more sharply in the observations than in the simulation. It continues to increase toward the center of the exhaust, surpassing *T*_||_ at the neutral plane. The total ion temperature shows the same sharp increase at the edge of the jet, while at the exhaust center it has a rather flat profile.

The observed average ion temperatures in the midexhaust ( $|B_{X}|<0.6B_{0}$, intervals 2 and 3) were $〈T_{i,||}〉=(0.24\pm 0.07)\;m_{p}V_{\text{A,in}}^{2}$, $〈T_{i,⊥}〉=(0.20\pm 0.05)\;m_{p}V_{\text{A,in}}^{2}$ and $〈T_{i,\text{tot}}〉=(0.21\pm 0.05)\;m_{p}V_{\text{A,in}}^{2}$. Performing similar calculations using the simulation data, we find $(0.23\pm 0.06)\;m_{p}V_{\text{A,in}}^{2}$, $(0.13\pm 0.02)\;m_{p}V_{\text{A,in}}^{2}$ and $(0.17\pm 0.01)\;m_{p}V_{\text{A,in}}^{2}$, respectively.

The observed 〈*T*_*i*,||_〉 and 〈*T*_*i*,⊥_〉 are within one standard deviation from each other; it is thus advisable not to compare the ratio of the means but to study the anisotropy itself. Part of the scatter in the observations is due to the slowly changing inflow conditions (increasing *B* and *n* variations): the lighter colored points observed later in time have on average lower temperatures. The rest of the variability is most likely due to spatial structures similar to those in the simulation (Figure 2) convecting past the spacecraft. This variability would be difficult to quantify using solar wind or magnetopause exhausts because they are generally crossed only once.

Let us briefly consider the observed partition of energy in the exhaust. The fraction of the inflowing Poynting flux turned into ion enthalpy flux can be calculated with [*Phan et al.*, 2014]

(1) $$〈\frac{\gamma }{\gamma -1}\Delta T_{i}/m_{p}V_{\text{A,in}}^{2}〉∼〈\frac{5}{2}T_{i}/m_{p}V_{A,\mathrm{in}}^{2}〉,$$

assuming *γ* = 5/3 and cold inflow. For the event considered here the ion enthalpy flux was thus (54 ± 13)%. The average jet speed in the same region was (0.57 ± 0.17)*V*_A,in_. The average fraction of the kinetic energy $\left(〈\frac{1}{2}V_{X}^{2}/V_{\text{A,in}}^{2}〉\right)$ was (18 ± 10)% of the available magnetic energy, clearly smaller than the ion enthalpy (ratio of 1 to 3).

Figures 3i and 3j show the behavior of the *α*_*i*_=(*β*_||*i*_−*β*_⊥*i*_)/2 parameter quantifying the ion temperature anisotropy. We see that the observed and simulated profiles match remarkably well. Outside of the exhaust plasma is rather isotropic (*α*_*i*_∼0). Moving into the exhaust, *α*_*i*_ becomes positive as *T*_*i*,||_ dominates. The firehose stability condition is given by *α*_tot_>1. We have not included the electrons as their contribution to *α*_tot_ would be small: the observed electrons were colder than the ions (by a factor of 4) and quite isotropic (*α*_e_∈[−0.2,0.5]), and the simulated electrons were also more isotropic than the ions (not shown). Clearly, around |*B*_*X*_|∈[0.1,0.5]*B*_0_, the jet is at times unstable. From Figure 2d we also see that the firehose unstable areas are nonspace filling which explains why there is a large spread of *α*_*i*_ values from ≪1 to ≫1 in Figure 3j.

The very center of the current sheet ( $|B_{X}|≲0.1B_{0}$) is dominated by negative *α*_*i*_ values, as *T*_⊥_>*T*_||_ both in the simulation and in the observations. This points to Speiser‐like ion motion and suggests that the observed field reversal region within the exhaust was thin (*κ* < 1). The spatial thickness of this negative *α*_*i*_ layer in the simulation is ∼1*d*_*i*_ (Figure 2d).

**Figure 4:**
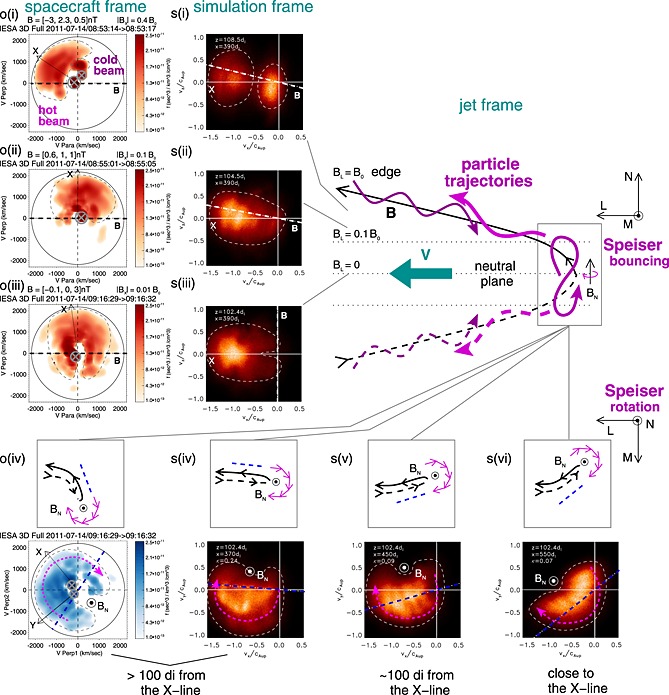
Observed (o) and simulated (s) distribution functions at different magnetic latitudes (i–iii) and in the midplane at different distances downstream of the X line (iv–vi). Panels o(i–iii) show cuts in the **V**‐**B** plane (the horizontal axis is parallel to local **B**, and the perpendicular axis contains the bulk velocity). Panel o(iv) shows a cut in the plane perpendicular to the magnetic field of the same distribution as o(iii). High values near the origin due to residual background counts are covered with gray ⊗ symbols. The black arrows note the *V*_X,GSM_ direction and the main features are sketched with black dashed curves. Panels s(i–iii) show simulated distributions in the **vx**‐**vz** plane. The thick white dashed line gives the local magnetic field orientation and the main features are highlighted with white dashed outlines. Panels s(iv–vi) show distributions in the **vx**‐**vy** plane. The main cartoon (top right) depicts the ion dynamics in the reconnection plane in the jet frame. Ions (magenta) stream in along the magnetic field (black) from both sides, and undergo Speiser‐like motion (rapid bouncing plus slow rotation around *B*_*N*_) in the field reversal region before escaping and streaming outwards. The dashed lines indicate field lines and trajectories below the neutral plane. The four insets accompanying the distributions o(iv) and s(iv–vi) illustrate how magnetic fluctuations affect the Speiser‐like motion by moving the tip of the field line loop in and out of the reconnection plane. The thick blue dashed line depicts the orientation of the tip determined by **B** just outside the neutral plane.

The temperature profiles can be understood in terms of the ion dynamics, shown in Figure 4. The observed ESA full mode distributions (o) were taken during intervals of steady **B** direction, as confirmed by higher than spin resolution measurements. From the edge of the exhaust to intermediate distances (o(i)), we see in the **V**‐**B** plane two counterstreaming beams that have a common **E** × **B** drift (in the spacecraft frame) and field‐aligned drifts that are in opposite directions. Distribution o(i) was taken below the midplane (*B*_*X*_∼−3 nT), so the incoming beam moving parallel to **B** is colder, and the beam moving away from the neutral plane antiparallel to **B** is hotter. Close to the neutral plane (o(ii), *B*_*X*_∼0.1*B*_0_), the two beams are indistinguishable, forming a distribution that is firehose unstable with *α*_*i*_∼4.

The third distribution (two different cuts o(iii) and o(vi)) was taken from the neutral plane (*B*_*X*_∼0.01*B*_0_), where we expect Speiser‐like motion of rapid bouncing in the direction normal to the plasma sheet combined with slow rotation around *B*_*N*_. In the **V**‐**B** plane (o(iii), red) we see ions moving up and down the magnetic field with a range of velocities perpendicular to it: The ions with smaller *v*_⊥_ have recently entered the exhaust. Those with large *v*_⊥_ (extending above the instrument energy range) probably originate closer to the X line and are hotter so that the gap between the upwards and downwards moving particles is no longer distinct. In the plane perpendicular to **B** (o(iv), blue) we see the crescent/horseshoe shape characteristic to Speiser‐like motion [e.g., *Nakamura et al.*, 1998; *Lottermoser et al.*, 1998]: the ions come into the field reversal region with a velocity oriented along the magnetic field outside of it (blue dashed line; see discussion below), rotate slowly around *B*_*N*_ (∼*B*_*Z*_) for about half a circle, and escape when their velocity is again aligned with the field outside.

We have also examined all the observed reduced mode distributions that fulfill the condition |*B*_*X*_|<0.1*B*_0_ and *α*_*i*_<0: there are 49 such distributions for 08:16–09:00 UT and 94 for 09:00–09:30 UT. More than thirty show Speiser‐like features similar to o(iii) and o(iv).

The observed ion behavior is reproduced by the simulation: Considering the **vx**‐**vz** plane and proceeding from the exhaust edge to the midplane, we see the two counterstreaming beams (s(i)) that merge into a firehose unstable distribution (s(ii), *α*_*i*_∼1.4). The midplane distribution s(iii) can be compared with o(iii) by rotating it by 90°, and it shows the same Speiser‐like bouncing. Note, however, that in the simulation *β*_in_ was larger than in the observations, so the inflowing beam in s(i) is wider and the gap at low energies in s(iii) is not as clear.

The simulation reveals how the orientation of the Speiser horseshoe on the plane perpendicular to **B** (the **vx**‐**vy** plane) depends on the magnetic field direction just outside the reversal region, i.e., mainly on *B*_*X*_ and *B*_*Y*_. For each simulated distribution s(iv–vi) we have calculated this **B** direction and drawn it on the cut (blue dashed lines). Close to the X line horseshoe's orientation is determined by the standard Hall field (s(vi)) [*Nakamura et al.*, 1998; *Drake et al.*, 2009]. Further downstream, the orientation of the tip of field line loop varies, as illustrated by the small cartoons, due to the large (∼0.5*B*_0_) *B*_*Y*_ fluctuations (Figures 2a and 3a and 3b) and so does the orientation of the crescent/horseshoe (s(iv–v)). The orientation of the observed horseshoe distribution (o(iv)) indicates that the tip was twisted in +*Y*_GSM_∼+*M* direction, which is consistent with the magnetic field measurements taken before the distribution ([−0.1, 0.5–1.0, 3] nT) showing positive *B*_*Y*_ below the neutral plane that is much larger than *B*_*X*_. The inferred field direction is sketched on o(iv).

## 4 Discussion and Conclusions

We have investigated the ion temperature anisotropy across a jet arising from reconnection of antiparallel magnetic fields in the midmagnetotail, far from the X line. Our results of high *T*_*i*,||_ near the edges of the exhaust are in agreement with *Hoshino et al.* [1997], who plotted *T*_total,||_/*T*_total,⊥_ against *B*_*X*_ for one year of Geotail's observations of hot and fast plasma flows. However, this approach using many events did not normalize the individual measurements to the lobe *B*_0_ and *V*_A,in_.

Figures 3i and 3j show that the firehose limit is greatly exceeded in parts of the jets. This somewhat surprising finding could indicate that the driving of the instability is much stronger (faster) than the growth of the instability [*Matteini et al.*, 2006; *Kunz et al.*, 2014]. A possible explanation is that the unstable parameter region (two counterstreaming beams) is continuously refilled as plasma enters into the exhaust along the exhaust boundaries (blue arrows in Figure 2b). The growth rate of firehose is around 10 ion gyroperiods, which at *B*_*X*_∼2 nT ∼0.25*B*_0_ is ∼300 s. It only takes about ∼50 s for an incoming ion at 0.1*V*_A,in_∼120 km/s to cross the estimated distance of 1 *R*_*E*_ from the exhaust boundary to the neutral plane. In ∼300 s the plasma in the jet with a speed ∼700 km/s also moves ∼30 *R*_*E*_∼200*d*_*i*,exhaust_ downstream. The situation is probably different in near tail, where the exhaust is wider and magnetic field is stronger; recently, *Wu et al.* [2013] reported more isotropic plasma in bursty bulk flows observed at *X* >− 14 *R*_*E*_ than at *X* <− 14 *R*_*E*_.

Speiser‐like, meandering ion motion at the neutral plane of the reconnection exhaust has been reported in many hybrid [e.g., *Nakamura et al.*, 1998; *Lottermoser et al.*, 1998; *Arzner and Scholer*, 2001; *Higashimori and Hoshino*, 2012] and PIC [e.g., *Drake et al.*, 2009; *Zenitani et al.*, 2013] simulations and within the ion diffusion region [*Nagai et al.*, 2015] and in a plasmoid event in the magnetotail [*Hoshino et al.*, 1998]. *Higashimori and Hoshino* [2012] found the Speiser‐like motion to be limited to within 70*d*_*i*_ from the X line, while *Lottermoser et al.* [1998]; *Arzner and Scholer* [2001] found it to persist up to ∼200*d*_*i*_ where the current sheet disrupted. In the simulation presented here the Speiser regime extends at least 220*d*_*i*_ downstream of the X line (*κ* < 1). This corresponds to the region where *T*_*i*,⊥_ is large at the midplane. Our observations agree with this simulation and indicate that such motion persists at large distances (>100*d*_*i*_) from the X line in the midmagnetotail.

Comparing the temperature increase observed in this magnetotail jet to magnetopause and solar wind reconnection jets, we find that the value 0.21 ± 0.05 (in terms of the fraction of energy released) is larger than the average of ∼0.13 reported in these other regions [*Phan et al.*, 2014; *Drake et al.*, 2009]. However, it is unclear whether this discrepancy is significant considering the uncertainty in the determination of the density (and thus the Alfvén speed) in the inflow (lobe) region in our event and the variability from event to event in the solar wind and magnetopause statistical studies. A similar statistical study in the magnetotail is needed. We also find that the observed temperatures in our event are higher than in the simulation, especially in the perpendicular direction. On the one hand, the simulation is 2.5D and *m*_*i*_/*m*_*e*_=25, and the inflow *β*_in_=0.2 compared to the observed $\beta_{\text{in}}≲0.05$. On the other hand, the exact lobe plasma conditions are challenging to measure. Finally, we note that although we have used a constant normalization to a fixed value of *V*_A,in_ in this study, from Figure 1 we can also see that the ion temperature variations over time show anticorrelation (correlation) with the density (jet speed). This supports the general conclusion that heating is controlled by the inflow region Alfvén speed.

In summary, we find good agreement between the ARTEMIS observations and the PIC simulation of ion heating in antiparallel, symmetric reconnection, far away (>100*d*_*i*_) from the X line. In quantitative terms, the mean total ion temperatures were similar: $(0.21\pm 0.05)m_{p}V_{\text{A,in}}^{2}$ for the observations and $(0.17\pm 0.01)m_{p}V_{\text{A,in}}^{2}$ for the simulation. *T*_*i*,||_ dominates near the exhaust boundary, and the distributions show a slow, cold beam and a hot, fast beam. The firehose condition is often greatly exceeded within patchy spatial regions at |*B*_*X*_|∈[0.1,0.5]*B*_0_, indicating that the driving caused by the reconnection geometry is stronger than the instability. *T*_*i*,⊥_ dominates at the neutral plane ( $B_{X}≲0.1B_{0}$). This corresponds to ions performing Speiser‐like meandering motion, despite being in the turbulent exhaust far away from the X line.

## Supporting information



Supporting Information S1
