# Supplementary material for: Ion temperature anisotropy across a magnetotail reconnection jet
Source: Geophys Res Lett. 2015 Sep 18;42(18):7239–47. doi: 10.1002/2015GL065168 (PMC4950132; doi:10.1002/2015GL065168)
Supplement: Supplementary file 1 — Supporting Information S1 [file GRL-42-7239-s001.pdf]

Supporting Information for "Ion temperature anisotropy across a magnetotail reconnection jet"

H. Hietala[1], J. F. Drake[2], T. D. Phan[3] J. P. Eastwood[1] and J. P. McFadden[3]

Contents of this file

1. Additional details of data, instruments and methods

Additional details of data, instruments and methods

We have analyzed DC magnetic field data from the Flux Gate Magnetometers (FGM; [*Auster et al.*, 2008]), plasma data from the Electrostatic Analyzers (ESA; [*McFadden et al.*, 2008]), high energy ion data from the Solid State Telescopes (SST; [*Angelopoulos*, 2008]), as well as the electric field and spacecraft potential measurements from the Electric Field Instrument (EFI; [*Bonnell et al.*, 2008]). The ESA reduced mode (24 energies, 50 angles) ion distributions were available every 3.6-second spin and the full mode (32 energies, 88 angles) ion distributions every 32 spins. The SST ion measurements (16 energies, 64 angles) were available every spin. To calculate the ion plasma moments we combined ESA and SST measurements by linearly extrapolating across the $\sim$16 keV gap from the ESA side. Low energy (< 100 eV) ion background counts due to energetic electrons were also removed. We use the Geocentric Solar Magnetospheric (GSM) coordinate system.

To map the spatial variations in the exhaust, we use the spin-resolution B_X as an estimate of the distance to the neutral plane together with the temperatures obtained by projecting the pressure tensor to the observed magnetic field direction. Similar maps can be recovered using the diagonalized temperatures and BS_X, where B is the observed magnetic field magnitude and $\mathbf{S}$ the principal axis of the pressure tensor. We exclude data points with $n < 0.01\,\mathrm{cm}^{-3}$.

For the observations we estimate the exhaust ion inertial length $d_{\mathrm{i,exhaust}}$ using the observed exhaust density. In the simulation the densities were normalized to the initial Harris sheet density n_{Harris}. (The background density in this run was $0.2\,n_{\mathrm{Harris}}$.) Consequently, the scales, e.g., in Figure 2 are shown relative to the ion inertial length d_{i} calculated with n_{Harris}. This initial plasma sheet d_{i} is $\sim 60\,\%$ of the simulation exhaust ion inertial length $d_{\mathrm{i,exhaust}}$.

References

Angelopoulos, V. (2008), The THEMIS mission, *Space Sci. Rev.*, *141*, 5–34, doi:10.1007/s11214-008-9336-1.

Auster, H. U., K. H. Glassmeier, W. Magnes, O. Aydogar, W. Baumjohann, D. Constantinescu, D. Fischer, K. H. Fornacon, E. Georgescu, P. Harvey, O. Hillenmaier, R. Kroth, M. Ludlam, Y. Narita, R. Nakamura, K. Okrafka, F. Plaschke, I. Richter, H. Schwarzl, B. Stoll, A. Valavanoglou, and M. Wiedemann (2008), The THEMIS Fluxgate Magnetometer, *Space Sci. Rev.*, *141*, 235–264, doi:10.1007/s11214-008-9365-9.

Bonnell, J. W., F. S. Mozer, G. T. Delory, A. J. Hull, R. E. Ergun, C. M. Cully, V. Angelopoulos, and P. R. Harvey (2008), The Electric Field Instrument (EFI) for THEMIS, *Space Sci. Rev.*, *141*, 303–341, doi:10.1007/s11214-008-9469-2.

McFadden, J. P., C. W. Carlson, D. Larson, M. Ludlam, R. Abiad, B. Elliott, P. Turin, M. Marckwordt, and V. Angelopoulos (2008), The THEMIS ESA Plasma Instrument and In-flight Calibration, *Space Sci. Rev.*, *141*, 277–302, doi: 10.1007/s11214-008-9440-2.

Corresponding author: H. Hietala, The Blackett Laboratory, Imperial College London, Prince Consort Road, London SW7 2AZ, UK. (h.hietala@imperial.ac.uk)

[1]The Blackett Laboratory, Imperial College, London SW7 2AZ, UK

[2]University of Maryland, USA

[3]Space Science Laboratory, University of California, Berkeley, USA

Copyright 2015 by the American Geophysical Union.
0094-8276/15/$5.00
